# Vector competence of *Aedes aegypti* populations from Kilifi and Nairobi for dengue 2 virus and the influence of temperature

**DOI:** 10.1186/1756-3305-7-435

**Published:** 2014-09-15

**Authors:** Edith Chepkorir, Joel Lutomiah, James Mutisya, Francis Mulwa, Konongoi Limbaso, Benedict Orindi, Zipporah Ng’ang’a, Rosemary Sang

**Affiliations:** International Centre of Insect Physiology and Ecology, P.O. Box 30772-00100, Nairobi, Kenya; Center for Virus Research, Kenya Medical Research Institute, P.O Box 54628-00200, Nairobi, Kenya; Jomo Kenyatta University of Agriculture and Technology, P.O. Box 62000-00200, Nairobi, Kenya

**Keywords:** *Aedes aegypti*, Vector competence, Temperature

## Abstract

**Background:**

Susceptibility of *Ae. aegypti* mosquito to dengue virus (DENV) varies geographically and can be influenced by climatic factors such as temperature, which affect the incidence, seasonality and distribution of vector-borne diseases. The first outbreak of dengue fever (DF) in Kenya occured in 1982 in the coastal towns of Malindi and Kilifi. Unlike Nairobi where no active dengue transmission has been reported, DF is currently re-emerging at the Coast causing major outbreaks. This study investigated the vector competence of *Ae. aegypti* populations from two urban areas, Kilifi (Coast of Kenya) and Nairobi (Central Kenya), for DEN-2 virus and the influence of temperature on the same.

**Methods:**

Four-day old adult female *Ae. aegypti* mosquitoes collected as eggs from the two sites were exposed to defibrinated sheep blood mixed with DEN-2 virus (10^5.08^ PFU/ml) using a membrane feeder. Half of the exposed mosquitoes were incubated at high temperature (30°C) and the other half at low temperature (26°C), and every 7 days up to day 21 post-infection 30% of the exposed mosquitoes were randomly picked, individually dissected, separated into abdomen and legs, and tested for midgut and disseminated infection, respectively, including virus quantification by plaque assay using Vero cells.

**Results:**

Nairobi mosquito populations exhibited significantly higher midgut infection rates (16.8%) compared to the Kilifi population (9%; p = 0.0001). Midgut infection rates among the populations varied with temperature levels with a significantly higher infection rate observed for Nairobi at high (21.3%) compared to low temperature (12.0%; p = 0.0037). Similarly, for the Kilifi population, a significantly higher infection rate was recorded at high (11.6%) relative to low temperature (6.8%; p = 0.0162). It is however, noteworthy that disseminated infection was higher among the Kilifi mosquito population (40.7%) than in Nairobi mosquitoes (10.3%; p < 0.0001).

**Conclusion:**

The findings show a clear inherent difference between the two populations in their ability to develop disseminated infection with high temperature having an added effect of enhancing vector competence. Therefore, the inherent difference among the two populations of *Ae. aegypti* coupled with prevailing ambient temperature could partly explain the distribution of dengue 2 virus between the Coastal and Nairobi regions in Kenya.

## Background

Dengue virus, a mosquito-borne virus belonging to the genus *Flavivirus* and family *Flaviviridae,* exists in 4 distinct serotypes (DEN 1-4) [1]. DENV constitutes a major public health concern, infecting millions of people per year in tropical and subtropical areas globally [2]. Dengue illness in humans presents with a wide spectrum of clinical manifestations, ranging from a flu-like Dengue Fever (DF), to the more severe Dengue Haemorrhagic Fever (DHF) and Dengue Shock Syndrome (DSS).

Dengue fever caused by all four serotypes has been on the increase since 1980, mostly affecting Asia, South America and the Caribbeans [3, 4]. Although there is limited documentation on the burden of Dengue in Africa [4] recent events suggest major re-emegence of the disease in Africa with outbreaks having been reported in East Africa (i.e South Sudan, Somalia, Kenya and Tanzania) [5] and West Africa (i.e Senegal, Central Africa Republic) [6]. In Kenya, the first outbreak of dengue fever caused by dengue virus 2 (DEN-2) occured in 1982 in the coastal towns of Malindi and Kilifi [7]. A serosurvey carried out in 2005 revealed the occurrence of dengue transmission in coastal and inland parts of Kenya [8]. In 2012, dengue outbreak was reported in Northern Kenya and subsequently in the coastal town of Mombasa (KEMRI lab reports 2012-2014) and is now viewed as a re-emerging public health problem in Kenya.

*Aedes aegypti* mosquito, the principal vector of DENV vector, originated in Africa and spread to tropical countries in the 17^th^ and 18^th^ centuries [9] *.* Urbanization is a major factor in facilitating the increase of *Aedes* mosquito species populations [1]. Accumulation of non-biodegradable, man-made containers used to store water in and around living areas provides the aquatic breeding environment required by these mosquitoes [10]. Based on these demographic changes and subsequent increases in *Aedes* mosquito populations, Appawu [11] predicted an increase in DENV transmission in Africa. It has been suggested that susceptibility of different strains of *Aedes* species and populations to DENV varies geographically affecting DENV distribution in Africa [12] possibly driven by inherent and climatic factors [13, 14].

Vector competence (VC) is the intrinsic permissiveness of an arthropod vector for infection, replication and transmission of a virus [15], mediated by the presence of several genetically determined barriers to viral transmission, including a midgut infection barrier (MIB) that prevents invasion and replication of the viruses and a midgut escape barrier (MEB) that prevents dissemination to other tissues [16]. These barriers are major determinants of vector competence to DENV during experimental infections [17]. The barriers also vary in prevalence in natural populations, leading to large intraspecific variation of *Ae. aegypti* vector competence and may determine the epidemiology of dengue viruses [16].

*Ae. aegypti* is widely distributed across Kenya [18] and the risk of dengue virus transmission is therefore likely to be equally widespread. Dengue outbreaks have been reported multiple times at the Kenyan coastal towns of Malindi, Kilifi [7] and most recently Mombasa (KEMRI lab reports 2012-2014), but in spite of the presence of *Ae. aegypti* in Nairobi DENV transmission has not been documented. Whether these outbreaks are related to the urbanization and the area being a tourist destination is unclear. Like the coastal towns, Nairobi the capital city of Kenya is a major international hub hosting the largest airport in the East African region, facilitating the influx of a large number of international travelers. To explain the differences in outbreak occurrence between Nairobi and the Coast, we hypothesized that the vectorial capacity of the *Ae. aegypti* populations between the two areas are different. While inherent differences between *Ae. aegypti* populations may exist, the contribution of differential ambient temperatures between these sites in driving the observed DEN transmission patterns has never been assessed. Therefore, this study sought to evaluate the vector competence of *Ae. aegypti* populations from Nairobi and Kilifi in laboratory experiments while incorporating the effect of prevailing ambient temperatures at these sites.

## Methods

### Ethical considerations

Scientific and ethical approval was obtained from the Kenya National Ethical Review Committee at the Kenya Medical Research Institute (protocol KEMRI/RES/7/3/1) dated 26^th^ September, 2012. Animal use approval was obtained from the KEMRI Animal Care and Use Committee (ACUC).

### Study area

**Figure 1:**
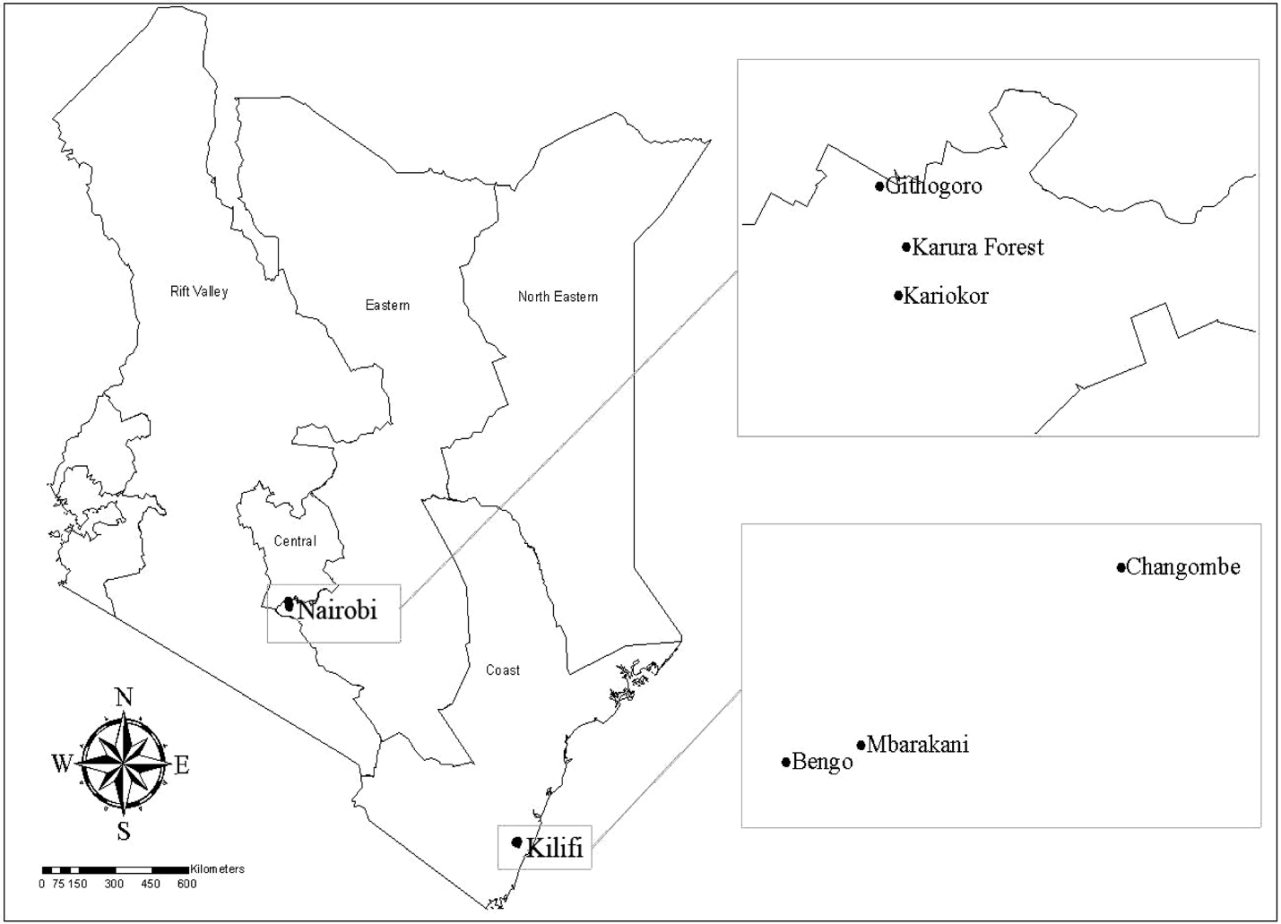
**Map showing the study area and the sites where** ***Ae. aegypti*** **eggs were collected.**

The study sites included the Coastal and Central parts of Kenya (Figure 1). At the coast, eggs of *Ae. aegypti* mosquitoes were collected from villages in Rabai in Kilifi County based on prior history of dengue circulation in the area [7]. Kilifi County (latitude of 3.63°S and a longitude of 39.85°E) has a mean daily temperature of 30°C, rainfall of approximately 88.25 mm and 82% relative humidity. Collections in Nairobi were from Karura on the outskirts of Nairobi (latitude of 1.28°S and a longitude of 36.81°E) with a mean daily temperature of 25°C, rainfall of approximately 85.35 mm and 70% relative humidity. The study adopted a laboratory-based experimental design.

### Mosquito egg collection

*Aedes aegypti* eggs were collected using oviposition cups (ovicups), lined with oviposition papers and half-filled with water. These were set at various points, at least 100 meters from each other, in the study area for 4 days with the Global Positioning System (GPS) coordinates of each ovicup point taken for geo-referencing. On day 4, all the ovicups were collected and the eggs transported to the biosafety level-2 (BSL-2) insectary at KEMRI in Nairobi, where they were dried on damp cotton wool to quiescent state [19], and stored in an air tight container at room temperature in the insectary.

### Mosquito rearing

Mosquitoes were reared in the KEMRI insectary, maintained at a temperature of 28°C and 80% relative humidity (RH), with a 12:12-hour (Light:Dark) photoperiod. As needed, several batches of eggs (F_0_) from Kilifi and Nairobi areas were dispensed in water on larval trays for hatching in a level 2 insectary. The larvae were fed on yeast mixed with tetramin fish food until they pupated. The pupae were collected every morning and put in a cup containing water. The cup with pupae was placed in a 1-gallon plastic cage with a netting material on top and allowed to develop into F_0_ adult mosquitoes. The emerging adults were knocked down by placing them in a -20°C freezer for one minute, then morphologically identified under a dissecting microcope using taxonomic keys of Edwards [20], to ensure that only *Ae. aegypti* mosquitoes were used in the subsequent experiments. Positively identified *Ae. aegypti* mosquitoes were returned to their experimental cages, blood fed on clean laboratory-bred mice and provided with oviposition papers to lay F_1_ eggs. The F_1_ eggs were hatched and reared as decribed above. Only adult female mosquitoes were used in the subsequent experiments [21].

### Dengue virus amplification

Dengue virus serotype 2 (DEN-2) which was isolated from a patient’s sample (Sample number: 008/01/2012) from the 2012 dengue outbreak in Mandera, Kenya, was used in the study. The virus was passaged in T-75 cm^2^ culture flask containing C6/36 cell lines (*Ae. albopictus* mosquito cell lines), grown in Dulbecco’s modified Eagle’s medium (DMEM), (GIBCO® Invitrogen corporation, Carlsbad, California), liquid (4.5 g/L D-glucose) without L-glutamine and sodium pyruvate, supplemented with 10% heat-inactivated fetal bovine serum (FBS), (Sigma-Aldrich, St. Louis, MO), 2% L-Glutamine (Sigma-Aldrich, St. Louis, MO), and 2% antibiotic/ antimycotic solution with 10,000 units penicillin, 10 mg streptomycin and 25 μg amphotericin B per ml (Sigma-Aldrich, St. Louis, MO) and incubated at 28°C in 5% CO_2_ over night. Confluent monolayer of C6/36 cells were inoculated with 600 μl of the dengue virus supernatant isolate and incubated for 1 hour with frequent agitation/rocking to allow for virus adsorption. The infected cells were maintained in DMEM supplemented with 2% FBS, 2% L-Glutamine and 2% antibiotic/antimycotic, incubated at 28°C in 5% CO_2_ and observed daily for cytopathic effect (CPE) for a period of 7 days. Once the CPE was observed to affect 80% of the monolayer, the flask was frozen overnight at -80°C, thawed on wet ice, then clarified by centrifugation at 3000 revolutions per minute for 10 minutes and the supernatant harvested by aliquoting into 1.5 ml cryovials. All the aliquots were stored at -80°C [22].

### Dengue virus quantification

Quantification of dengue virus was performed by plaque assay. Ten fold serial dilutions of the amplified DENV was carried out and inoculated in 6 well plates containing confluent Vero monolayers as described by Gargan *et al.,* [23]. This was grown in Minimum Essential Medium Eagle (MEM), (Sigma-Aldrich, St. Louis, MO) with Earle’s salts and reduced NaHCO_3_, supplemented with 10% heat-inactivated fetal bovine serum (FBS), (Sigma-Aldrich, St. Louis, MO), 2% L-Glutamine (Sigma-Aldrich, St. Louis, MO), and 2% antibiotic/antimycotic solution with 10,000 units penicillin, 10 mg streptomycin and 25 μg amphotericin B per ml (Sigma-Aldrich, St. Louis, MO) and incubated at 37°C in 5% CO_2_ over night. Each well was inoculated with 100 μl of virus dilution, incubated for 1 hour with frequent agitation/rocking to allow adsorption. The infected cells were maintained using 2% methylcellulose mixed with 2X MEM (GIBCO® Invitrogen corporation, Carlsbad, California) and incubated at 37°C with 5% CO_2_ for 9 days then fixed for 1 hour with 10% formalin, stained for 2 hours with 0.5% crystal violet and the plaques counted and calculated to quantify the virus using the formula [23]:


where d is the dilution factor and V is the volume of diluted virus added to the well.

## Susceptibility studies

### Mosquito infection

An infectious blood meal was prepared by mixing DEN-2 virus stock with a virus titer of 10^5.08^ plaque-forming units (PFU)/ml and defibrinated sheep blood, in a ratio of 1:1, to end up with 10^3.03^ PFU/ml virus concentration. The virus/blood mixture was put in membrane feeders covered with a freshly prepared mouse skin [24], and maintained using the Hemotek system which employs an electric heating element to maintain the temperature of the blood meal at 35°C ± 1°C [25]. Four-day-old adult female mosquitoes were allowed to feed on the infectious blood meal through the mouse skin for 1 hour. After feeding, fully engorged mosquitoes were selected and put in secured cages, where they were maintained on 8% glucose for 7 to 21 days at set temperatures and 80% relative humidity. The first set of experiments involved maintaining both Kilifi and Nairobi exposed mosquitoes to temperatures set at an average of 26°C (i.e. similar to Nairobi temperature conditions). The second set of experiments involved maintaining another group of exposed mosquitoes from both sites to temperatures set at an average of 30°C (i.e. similar to Kilifi temperature conditions). Mortality was monitored in the cages by removing and counting dead mosquitoes daily [25].

### Test for infection and dissemination rates of Dengue-2 virus

After every 7 days up to day 21 of incubation, 30% of live exposed mosquitoes were randomly picked and each dissected to separate the abdomens and legs. Individual abdomens and legs were placed separately in 1.5 ml eppendorf tubes containing 500 μl of homogenizing media (HM), consisting of MEM, supplemented with 15% FBS, 2% L-Glutamine, and 2% antibiotic/ antimycotic. The individual abdomens were homogenised using plastic grinders and the supernatant diluted in 10 fold serial dilutions. The dilutions were inoculated in confluent Vero cell lines in 12-well plates, grown in MEM, supplemented with 10% FBS, 2% L-Glutamine and 2% antibiotic/antimycotic. The infected cell monolayers were then overlaid with methylcellulose supplemented with 2% FBS, 2% L-Glutamine and 2% antibiotic/ antimycotic and incubated at 37°C in 5% CO_2_. On day 9, plates were fixed for 1 hour with 10% formalin, and stained for 2 hours with 0.5% crystal violet, washed on running tap water, dried overnight and the plaques observed on a light box. For each positive abdomen, corresponding legs were homogenised and their infection status determined as described above for the abdomens. Plaques were counted and calculated to determine the viral titer [26].

### Data analysis

Analyses were performed using R version 2.15.1 [27]. To gain some insight into the dataset, descriptive statistics and graphical displays were used. Data on *Ae. aegypti* mosquito infection and dissemination rates at the two temperature levels and/or sites were compared using Chi square (and Fisher’s exact) test at 5% significance level and confidence intervals (CIs) for the proportions estimated [28, 29].

## Results

### Susceptibility to dengue- 2 virus infection in Nairobi and Kilifi mosquitoes

**Figure 2:**
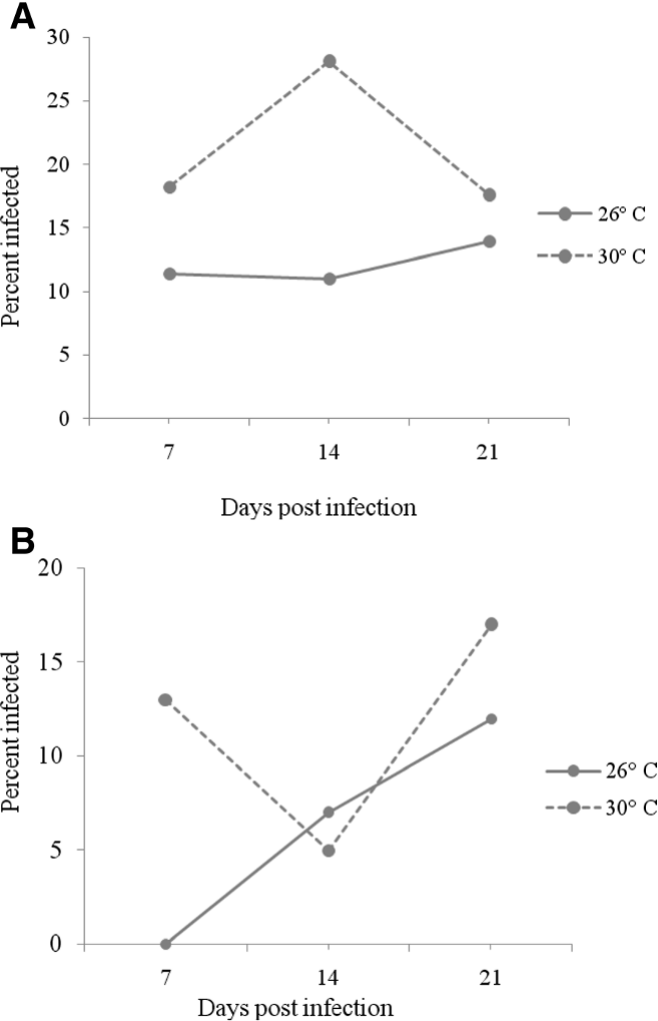
**Proportion of Nairobi (A) and Kilifi (B)** ***Ae. aegypti*** **infected at day 7, 14 and 21 post-infection at each temperature level.**

Dengue 2 virus infection rates were measured in a total of 517 *Ae. aegypti* female mosquitoes in three replicates from Nairobi region (249 in low and 268 in high temperature regimes). Of these, 87 mosquitoes (16.8%; 95% CI: 13.7-20.20.3%) had DEN-2 infection in the midgut. The proportion of Nairobi mosquitoes with midgut infection was significantly higher in high temperature (21.3%) than at low temperature (12.0%; p = 0.0037). Figure 2A presents the percentage of infected mosquitoes at day 7, 14 and 21 post-infection for each temperature level with the highest infection recorded on day 14 post-infection at a high temperature of 30°C.

Kilifi mosquito population infection rates were measured in a total of 600 *Ae. aegypti* female mosquitoes in three replicates (300 in low and 300 in high temperature regimes). Of these, 54 mosquitoes (9%; 95% CI: 6.8-11.6%) developed midgut infection, with a significantly higher proportion recorded at high temperature (11.6%) relative to low temperature (6.8%; p = 0.0162). The percent of infected mosquitoes at day 7, 14 and 21 post-infection for each temperature level is presented in Figure 2B. There were low infection rates at 14 days post-infection under high temperature, with the highest infection at day 21 for both temperature levels.

**Figure 3:**
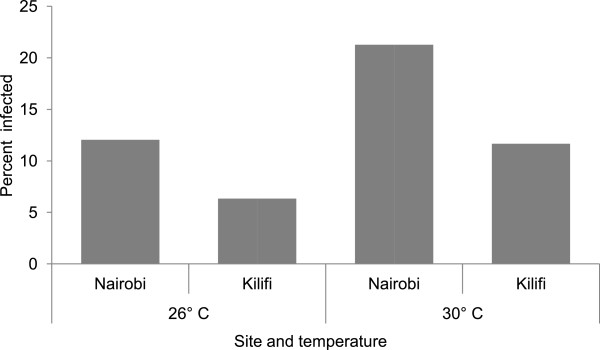
**Percent mosquitoes infected per site at each temperature level.**

Overall data for both Nairobi and Kilifi mosquito populations showed that 141 of the 1117 mosquitoes (12.62%; 95% CI: 10.73-14.71%) had midgut infection with a significantly higher proportion recorded in Nairobi (16.8%) than Kilifi (9%; χ^2^ = 14.73, df = 1, p = 0.0001). Analysis from both experiments showed that mosquito infection was significantly influenced by temperature with higher infections recorded for mosquito populations from Nairobi relative to Kilifi at both temperature levels (Figure 3).

### Dissemination of dengue-2 virus in Nairobi and Kilifi mosquitoes

**Figure 4:**
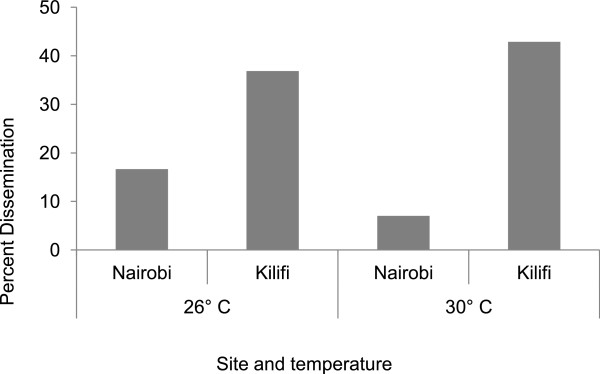
**Percent of infected mosquitoes with disseminated infection per site at each temperature level.**

The proportion of mosquitoes with disseminated infection was significantly higher for Kilifi mosquito population (40.7%; 95% CI: 27.6-55.0%) relative to that of Nairobi population (10.3%; 95% CI: 4.8-18.7%; p < 0.0001). This was evident at both the low and high temperature levels (Figure 4). Figure 4 also shows that, while the highest dissemination was observed for Kilifi population at the high temperatures, the lowest was recorded for Nairobi mosquito population, a finding which contrasts the pattern observed with midgut infection rates, in which the highest rates were observed in the Nairobi mosquitoes.

The results further showed that, for the Nairobi mosquito population, a higher disseminated infection was recorded at low (16.7%, n = 30) relative to high temperature (7.02%, n = 57), although the difference was not significant (p = 0.2648). Our findings therefore, revealed a negative association between temperature and the dissemination of DEN-2 virus by the Nairobi mosquito population. In contrast for Kilifi mosquito population, a lower dissemination rate was recorded at low (36.84%) compared to at high temperature (42.86%), although the difference was not significant (χ^2^ = 0.0195, df = 1, p = 0.889).

## Discussion

There are a number of factors that contribute to the vectorial capacity of a mosquito for an arbovirus, including mosquito survival, density, proportion of infected mosquitoes that are feeding, length of extrinsic incubation period, vector susceptibility to the virus, and density of susceptible hosts [30]. We investigated *Ae. aegypti* vector susceptibility to midgut infection and ability to disseminate dengue-2 virus, as a measure of the extent of adaptation of a virus to the vector and its potential to transmit it. However, the full competence of a vector is established by both its ability to become infected and to transmit the virus [26]. This parameter gives vector competence its epidemiologic importance. In this study, transmission potential was estimated from dissemination rates because previous studies have suggested that mosquitoes are capable of transmitting DENV as long as the virus is able to disseminate from the midgut into the hemocoel, subsequently finding its way to the salivary glands for transmission [31, 32]. An assumption was made based on these previous studies that mosquitoes that have a disseminated infection were capable of transmitting.

This study constitutes the first initial effort to understand the role of *Ae. aegypti* in driving dengue transmission in Kenya, to assess the risk of further spread of the disease by analyzing the vector competence of *Ae. aegypti* populations from parts of Kenya and the influence of environmental factors such as temperature, that prevail in outbreak hotspots and other areas of potential risk. The overall findings from this study demonstrate that the Nairobi *Ae. aegypti* population is a relatively inefficient vector for DEN-2 virus compared to that from Kilifi with the Nairobi population depicting high infection, but low dissemination rates in both low and high temperature settings. These findings suggest a weak midgut infection barrier (MIB) and a strong midgut escape barrier (MEB) for the Nairobi population and a moderate MIB, but weak MEB for the Kilifi population. The Kilifi *Ae. aegypti* population demonstrated inherent capacity to be more efficient in Dengue-2 virus transmission than the Nairobi population. Overall, the vector competence is considered as being inversely proportional to the level of MIB and MEB in the mosquito population: strong MIBs and MEBs reduce the potential for infection and dissemination and eventually transmission by the vector [17].

Geographic variation in *Ae. aegypti* populations to DENV susceptibility has been reported in various studies [31, 33]. A study done by Moncayo [30] on populations from various geographical locations showed that *Ae. aegypti* from Galveston, Texas, were more susceptible than those from Bolivia, but were less susceptible than mosquitoes from Thailand. Similar observations were made by Bennett [17] on *Ae. aegypti* collections from various locations in Mexico which differed significantly in their midgut susceptibility to infection. Our findings are similar to these studies’, because the midgut and disseminated infection rates differed significantly with *Ae. aegypti* populations collected from the two different sites, even after exposure to the same conditions in the laboratory.

Studies have also shown that temperature is one of the most important factors affecting biological processes of mosquitoes including their interaction with viruses [34, 35]. Our results demonstrate a significantly higher infection rate at high temperatures for mosquitoes from both Nairobi and Kilifi, which is consistent with results by Watts [34]. Although disseminated infection for many mosquito-borne viruses is known to be affected by temperature, this was not clearly observed in this study, but transmission of an arthropod-borne pathogen is only possible where there is disseminated infection [32].

*Aedes aegypti* is reported to have originated in Africa, adapted to the peridomestic environment of African villages before being exported to America by the slave trade and elsewhere by commercial transportation [36]. According to studies carried out by Moore [37], populations of *Ae. aegypti* outside Africa consist of mosquitoes arising from one of two ancestral clades. One clade is basal and is primarily associated with West Africa while the second arises from the first and contains primarily mosquitoes from East Africa. Mombasa at the Kenyan Coast is the second largest city of Kenya and a major port. It could therefore serve as an entry and exit point for this mosquito species, through human commerce. Apart from the favorable temperature at the coast, coastal mosquito populations may be genetically similar to those from the Asian continent due to the frequent arrivals of shipping vessels or activities between these regions that could provide opportunities for introduction of new Asian *Ae. aegypti* populations [37].

Whether the observed inherent difference in the competence to the virus between the *Aedes* populations from both sites is related to their genetics is unclear. It is known that *Aedes aegypti* exists in two forms or subspecies- *Aedes aegypti aegypti* and *Aedes aegypti formosus* [38–40]. While acknowledging the taxonomic difficulty in distinguishing these forms apart based on morphology [41], it may be worthwhile to understand in future studies how the populations between the sites compare genetically.

Environmental conditions play a major role in infection of viruses. High environmental temperature increases virus multiplication to high titers and reduces the extrinsic incubation period for the virus to be established within the vector [35]; get to the salivary glands after which it can be transmitted to another host [34]. Relative humidity on the other hand enhances mosquito survival allowing it to survive long enough for virus extrinsic incubation period to be complete allowing for virus transmission. Kilifi County experiences high annual temperature, rainfall and relative humidity as compared to Nairobi County which could explain why there have been several dengue fever outbreaks in the region. Data presented here reveals that the infection rates of DEN-2 virus in *Ae. aegypti* were significantly higher at high temperature regimes for both mosquito populations, suggesting a potentially significant role of temperature in the dynamics of DENV transmission. Both Kilifi and Nairobi *Ae. aegypti* populations were incubated at conditions that mimic the environmental conditions of both sites, but still low disseminated infection was observed in the Nairobi population. Like several earlier studies [34, 35], our findings highlight the need for a better mechanistic understanding of the environmental determinants of vector pathogen interactions.

## Conclusions

The findings show that both mosquito populations are susceptible to dengue-2 virus, with only Kilifi population supporting disseminated infection and transmission. The findings suggest an inefficient transmission ability of DEN-2 virus by the Nairobi *Ae. aegypti* population. This could explain why there has been no evidence of active dengue transmission in Nairobi despite reported cases of dengue in health facilities usually from individuals who have travelled from Mombasa (coast) and/or Mandera (North-eastern Kenya) where outbreaks have been reported. Environmental temperature also has a significant effect on the vector competence as evidenced from our results, which further explains ready transmission of dengue in Mombasa where mean temperatures are higher than Nairobi. Variation in vector competence among the populations of *Ae. aegypti* examined may help explain the distribution and spread of dengue fever. As the impact of climate change leads to increasing temperatures, spread of the virus through local vectors into susceptible host populations becomes likely. One of the limitations of this study is that it did not distinguish between *Ae. aegypti aegypti* and *Ae. aegypti formosus.* Therefore, it would be worthwhile for future studies to investigate how the two populations of this vector compare genetically in order to shed light on the observed inherent differences between the two populations in transmitting dengue-2 virus.
